# A new species of genus *Fejervarya* (Anura: Dicroglossidae) from northern Thailand

**DOI:** 10.13918/j.issn.2095-8137.2016.6.327

**Published:** 2016-11-18

**Authors:** Chatmongkon SUWANNAPOOM, Zhi-Yong YUAN, Nikolay A. POYARKOV Jr., Fang YAN, Somboon KAMTAEJA, Robert W. MURPHY, Jing CHE

**Affiliations:** ^1^State Key Laboratory of Genetic Resources and Evolution, Kunming Institute of Zoology, Chinese Academy of Sciences, Kunming Yunnan 650223, China; ^2^Division of Fishery, School of Agriculture and Natural Resources, University of Phayao, Phayao 56000, Thailand; ^3^College of Forestry, Southwest Forestry University, Kunming Yunnan 650224, China; ^4^Southeast Asia Biodiversity Research Institute, Chinese Academy of Sciences, Mengla Yunnan 666303, China; ^5^Department of Vertebrate Zoology, Biological Faculty, Lomonosov Moscow State University, Leninskiye Gory Moscow 119991, Russia; ^6^Joint Russian-Vietnamese Tropical Research and Technological Center Under the A.N. Severtsov Institute of Ecology and Evolution RAS, South Branch, Ho Chi Minh City, Vietnam; ^7^Faculty of Education, Chiang Rai Rajabhat University, Chiang Rai 57100, Thailand; ^8^Centre for Biodiversity and Conservation Biology, Royal Ontario Museum, Toronto M5S 2C6, Canada

**Keywords:** Phylogeny, Mitochondrial DNA, 16S rRNA, Chiang Mai Province, Cryptic species, Fejervarya chiangmaiensis ****sp. nov****

## Abstract

We describe a new species of frog in the dicroglossid genus *Fejervarya* from Ban Monjong, Omkoi District, Chiang Mai Province, northern Thailand. Analysis of DNA sequence data from the mitochondrial gene 16S, advertisement calls, and morphological distinctiveness support recognition of the new species. Matrilineal genealogy suggests that the new population from Chiang Mai is a sister taxon to the South Asian clade that includes *F. syhadrensis, F. granosa*, and *F. pierrei*. The new species, *Fejervarya chiangmaiensis* ****sp. nov**.**, differs morphologically from its congeners by its relatively small body size and proportions and the presence of dorsal warts and dermal ridges. Discovery of this new species indicates that the biodiversity of amphibians in this region remains underestimated.

## INTRODUCTION

The cricket frogs *Fejervarya* Bolkay currently contain 40 species (Frost, 2016), most of which occur in East and Southeast Asia, to the Indian subcontinent, including Sri Lanka, and further to Pakistan, Nepal, and Bangladesh (Dinesh et al., 2015). This genus comprises two reciprocally monophyletic species groups: (1) South Asian group; and (2) East and Southeast Asian group (Dinesh et al., 2015). Seven species within these two groups occur in Thailand (Frost, 2016), including *Fejervarya andamanensis* (Stoliczk, 1870); *Fejervarya cancrivora* (Gravenhorst, 1829); *Fejervarya limnocharis* (Gravenhorst, 1829); *Fejervarya moodiei* (Taylor, 1920); *Fejervarya multistriata* (Hallowell, 1861); *Fejervarya orissaensis* (Dutta, 1997); and, *Fejervarya triora* (Stuart et al., 2006). Except for *F*. *andamanensis*, which assigns to the South Asian group, all other Thai species assign to the East and Southeast Asian group (Frost, 2016). Several molecular studies have suggested that *F*. *limnocharis* from this region might represent an unnamed species (Dinesh et al., 2015; Kotaki et al., 2010; Kuramoto et al., 2007), such as, *F*. sp. hp3 from Pilok, Thailand and *F*. sp. hp2 from Bankok, Thailand (Kotaki et al., 2010). However, morphological characteristics of specimens from these regions have not been examined in detail. A closer inspection of many of these species is necessary to better understand and effectively manage the amphibian biodiversity in Thailand. 

Our recent fieldwork in Thailand resulted in the discovery of a new population of *Fejervarya*. To clarify its phylogenetic relationships with other species of *Fejervarya*, we reconstructed a matrilineal genealogy for the genus using mitochondrial DNA (mtDNA) sequence data from the ribosomal RNA gene 16S, which is widely recognized as a useful genetic marker for amphibian systematics (Vences et al., 2005a, b ). Our genealogy provides evidence for the phylogenetic placement of the new species. In addition, we examined the major morphological characters and acoustic data traditionally used in dicroglossid frogs. Based on these data, we describe the population as a new species of *Fejervarya*. 

## MATERIALS AND METHODS

### Sampling

**Figure 1:**
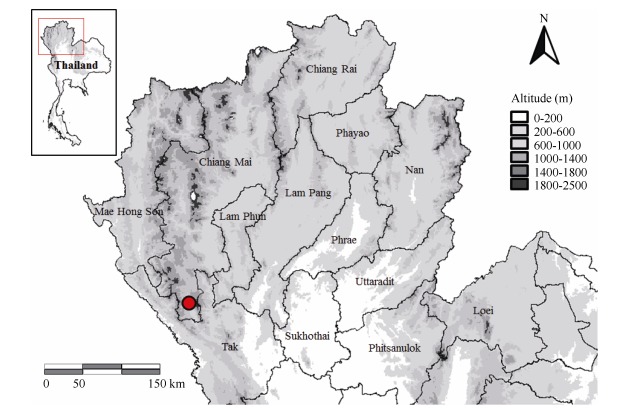
Known distribution of *Fejervarya chiangmaiensis* **sp. nov**. from northern Thailand, Omkoi District, Chiang Mai Province (red cycle=type locality)

Fieldwork was conducted in the vicinity of *Monjong village, Omkoi* District, * Chiang Mai Province, Thailand* (Figure 1) from June to September 2013. Twelve adult male frogs were collected in the field and photographed *in situ*. Specimens were euthanized using benzocaine after extraction of liver tissue, which was stored in 95% ethanol. The voucher specimens were fixed with 10% formalin and later stored in 70% ethanol. All specimens were deposited in the herpetological collection of the Museum of the Kunming Institute of Zoology (KIZ), Chinese Academy of Sciences (CAS). The protocols for collection of specimens in this study were approved by the Animal Ethics Committee of the KIZ, CAS. 

### Molecular methods

Total genomic DNA was extracted from tissue samples using standard phenol-chloroform protocols (Sambrook et al., 1989). One fragment of mtDNA encompassing the 16S rRNA gene (16S) was amplified using primers 16Sar: 5'-CGCCTGTTTAYC AAAAACAT-3' and 16Sbr: 5'-CCGGTYTGAACTCAGATCAY GT-3' from Kocher et al. (1989). Amplification was performed in a 25 µL volume reaction with the following procedure: initial denaturation at 95℃ for 5 min, 35 cycles of denaturation at 95℃ for 1 min, annealing at 55℃ for 1 min, extension at 72℃ for 1 min, and a final extension at 72℃ for 10 min. The PCR products were purified with a Gel Extraction Mini Kit (Watson Biotechnologies, Shanghai, China). All sequencing was conducted on a ABI PRISM 3730 automated sequencer (Applied Biosystems, Foster City, CA, USA) at KIZ, CAS. All individuals were sequenced in both directions. 

### Phylogenetic analysis

Sequences were examined for quality of signal and confirmed for complementarity using DNASTAR 5.0 (DNASTAR Inc., Madison, WI, USA). As the very small body size of the new specimens was most similar to that of species from the South Asian *Fejervarya* group, we chose *F*. *limnocharis*, *F*. *triora*, and *Limnonectes fujianensis* as outgroups based on Kotaki et al.(2008, 2010). Available sequences for these species were downloaded from GenBank as outgroups (Table 1). All mtDNA sequences were aligned using MUSCLE (Edgar, 2004). 

**Table 1:** Specimens corresponding to genetic samples included in phylogenetic analysis, their localities, and GenBank accession numbers

Species	Museum Cat. No.	Locality	GenBank Accession No.	Source
*Ingroup*				
*F. chiangmaiensis* ****sp. nov**.**	KIZ024057	Thailand: Chiang Mai; Omkoi	KX834135	This study
*F. chiangmaiensis* ****sp. nov**.**	KIZ024126	Thailand: Chiang Mai; Omkoi	KX834136	This study
*F. sahyadris*	RBRL 050714-02	India: Aralam	AB530605	Hasan et al. (2014)
*F. syhadrensis*	-	Sri Lanka	AY141843	Meegaskumbura et al. (2002)
*F. gomantaki*	CESF 2295	India: Goa	KR78086	Dinesh et al. (2015)
*F. granosa*	-	India: Mudigere	AB488895	Kotaki et al. (2010)
*F. pierrei*	-	Nepal: Chitwan	AB488888	Kotaki et al. (2010)
*F. kudremukhensis*	-	India: Kudremukh	AB488898	Kotaki et al. (2010)
*F.* cf. *nilagirica*	-	India: Western Ghats; Kudremukh	AB167949	Kurabayashi et al. (2005)
*F.* cf. *syhadrensis*	-	India: Karnool	AB488893	Kotaki et al. (2010)
*F. caperata*	-	India: Mudigere	AB488894	Kotaki et al. (2010)
*F. greenii*	-	Sri Lanka: Hakgala	AB488891	Kotaki et al. (2010)
*F. kirtisinghei*	MNHN 2000.620	Sri Lanka: Laggalla	AY014380	Kosuch et al. (2001)
*F. rufescens*	030526-03	India: Western Ghats; Mangalore	AB167945	Kurabayashi et al. (2005)
*F.* cf. *brevipalmata*	030607-01	India: Western Ghats, Madikeri	AB167946	Kurabayashi et al. (2005)
*F. mudduraja*	-	India: Madikeri	AB488896	Kotaki et al. (2010)
*F. keralensis*	WII:3263	India	JX573181	Unpublished
*F. limnocharis*	-	Indonesia: Java	AB277302	Kotaki et al. (2008)
*F. triora*	-	Thailand: Ubon Ratchatani	AB488883	Kotaki et al. (2010)
*Outgroup*				
*Limnonectes fujianensis*	-	China	AF315152	Unpublished

"-" denotes no museum Cat. No.

Phylogenetic reconstructions were executed using maximum parsimony (MP), maximum likelihood (ML), and Bayesian inference (BI). Character-based MP analyses were conducted using PAUP* v4.0b10 (Swofford, 2003). Full heuristic tree searches with tree bisection-reconnection were executed for 1000 replications. Bootstrap support (BS) for the MP tree involved 1 000 pseudoreplicates (Felsenstein, 1985). The ML analyses were performed with RAxML v7.0.4 (Stamatakis et al., 2008) using the Gamma model of rate heterogeneity option. Nodal support was estimated using 1 000 BS pseudoreplicates. For BI, the best-fit model of DNA sequence evolution was chosen using MrModeltest v2.3 (Nylander, 2004) under the Akaike information criterion. The GTR+I+G model was selected and used to generate a BI tree using MrBayes v3.1.2 (Ronquist & Huelsenbeck, 2003). Analyses were run for five million generations using four chains while sampling one of every 1000 tree generations and discarding the first 25% as burn-in. Log-likelihood scores were tracked to assure stationarity. 

Genetic distances among taxa were calculated using the Kimura 2-parameter model in MEGA 5. The matrilineal genealogy was assumed to reflect the phylogenetic relationships of the species. We considered tree nodes with bootstrap values 70% or greater and posterior probabilities values over 0.95 as sufficiently resolved, those between 75% and 50% (0.95 and 0.90 for BI) as tendencies, and those below 50% (0.90 for BI) as non-resolved (Huelsenbeck & Hillis, 1993). 

### Morphology

Measurements were made with digital calipers to the nearest 0.1 mm. Twenty morphometric characters of post metamorphic individuals were as per Matsui (1984) as follows: (1) snout-vent length (SVL); (2) head length (HL); (3) snout-nostril length (S-NL); (4) nostril-eye length (N-EL); (5) snout length (SL); (6) eye length (EL); (7) tympanum-eye distance (T-ED); (8) head width (HW); (9) internarial distance (IND); (10) interorbital distance (IOD); (11) upper eyelid width (UEW); (12) forelimb length (FLL); (13) lower arm length (LAL); (14) first finger length (FFL); (15) hindlimb length (HLL); (16) tibia length (TL); (17) foot length (FL); and (18) inner metatar sal tubercle length (IMTL). Additionally, we also measured (19) finger length (Ⅰ-Ⅳ FL) and (20) toe length (Ⅰ-Ⅴ TOEL). Toe-webbing states followed Savage (1975). 

We obtained comparative morphological data from museum specimens (Table 3), photographs of these specimens in life, and previously published literature: Jerdon, 1853; Günther, 1859, 1869; Peters, 1871; Boulenger, 1905; Annandale, 1919; Rao, 1922, 1937; Smith, 1930; Taylor, 1962; Inger, 1966; Dubois, 1975, 1984; Pillai, 1979; Manamendra-Arachchi & Gabadage, 1996; Dutta, 1997; Manthey & Grossmann, 1997; Dubois et al., 2001; Stuart et al., 2006; Orlov & Ananjeva, 2007; Matsui et al., 2007; Kuramoto et al., 2007; Ohler et al., 2009; Djong et al., 2011; Howlader, 2011a, b ; and Purkayastha & Matsui, 2012. Due to the high level of cryptic diversity within *Fejervarya*, we relied on examination of topotypic material and/or original descriptions of species when available. 

### Acoustics

Advertisement calls of the newly collected population were recorded *in situ* on 30 June, 2013, from 2200h to 2330h using a digital recorder (EDIROL R-09, Roland, Swansea, UK) with built-in microphone (frequency responses of 20-22.000 Hz). Files were recorded as 16-bit WAV files at a sampling frequency of 44.1 kHz and 22 advertisement calls from a single individual were recorded. The ambient temperature (26℃) was measured with a digital thermometer immediately after recording. Total duration of the recording was 3.0 s. We generated sound spectrograms of all field recordings using Syrinx-PC sound analysis software (J. Burt, Seattle, WA, USA) with the following settings: FFT size 512 samples and Hanning FFT window for spectrograms and power spectra, with FFT samples overlapping 75% for spectrograms. Comparative advertisement call characters for dicroglossids were taken from Kuramoto et al. (2007), Ohler et al. (2009), and Purkayastha & Matsui (2012). 

## RESULTS AND DISCUSSION

**Figure 2:**
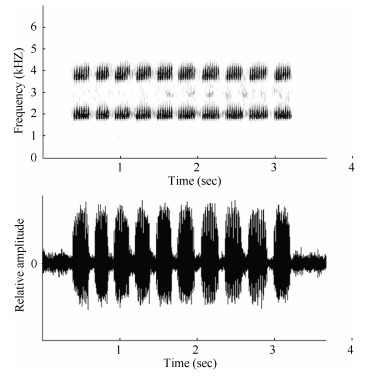
Sonogram and oscillogram of a *Fejervarya chiangmaiensis* **sp. nov**. call recorded on 30 June, 2013, at an agricultural farm in Ban Monjong, Omkoi District, Chiang Mai Province, Thailand

Morphological measurements and variations are summarized in Table 3. This species had both short and long calls, though the latter were not always emitted (Figure 2). The shorter call consisted of a series of pulsed notes. Each of these notes lasted 3.0±0.4 s and was composed of 9-12 pulses/call (average 11.2±1.8). The note interval was 1.81±0.598 s, the dominant frequency was 2.0±0.03 kHz, and the second harmonic was about 3 857±0.036 kHz. The call had a slight frequency modulation.

**Figure 3:**
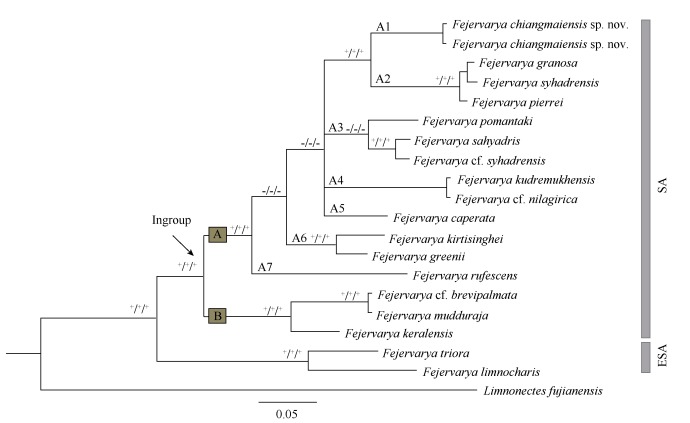
Matrilineal relationships among species of *Fejervarya* inferred from mtDNA 16S rRNA

All unique *de novo* sequences were deposited in GenBank under accession numbers KX834135 and KX834136. Sequencing generated a total of 700 base pairs (bp) of 16S rRNA data, among which 496 positions were potentially parsimony-informative. The ML, MP, and BI analyses produced similar topologies. Monophyly of the South Asian group of *Fejervarya* was recovered (Figure 3). Within these frogs, 16 species occurred in strongly supported matrilines A and B (Figure 3). Matriline A contained seven matrilines A1-7 (Figure 3), but their relationships were generally poorly resolved. Sub-matriline A1 contained the new population from Chiang Mai, which was the sister group of sub-matriline A2, which included *F*. *syhadrensis*, *F*. *granosa*, and *F*. *pierrei*. *Fejervarya sahyadris* formed a sister relationship with *F*. cf. *syhadrensis* within sub-matriline A3. Sub-matriline A4 included *F*. *kudremukhensis* and *F*. cf. *nilagirica*. Sub-matriline A5 included *F*. *caperata*. Sub-matriline A6 included *F*. *greenii* and *F*. *kirtisinghei*. Sub-matriline A7 included *F*. *rufescens*. Matriline B was comprised of *F*. *brevipalmata*, *F*. *murthii*, and *F*. *keralensis*.

The genetic distances among the 13 South Asian species ranged from 0.4% to 14.8% (Table 2). Interspecific genetic distances between the newly discovered Chiang Mai matriline A1 and members of sub-matriline A2 from Sri Lanka, India, and Nepal ranged from 6.0% to 6.7%. The new species differed from *F*. cf. *syhadrensis* and *F*. *sahyadris* (both within matriline A2) from India by 8.8 and 9.0%, respectively. The new species differed from *F*. *caperata* from India (matriline A4) by 9.2%, *F*. *kudremukhensis* and *F*. *nilagirica* (matriline A5) from India by 9.0%, *F*. *greenii* and *F*. *kirtisinghei* from Sri Lanka (matriline A6) by 9.7% and 9.8%, respectively, and *F*. *rufescens* from matriline A7 by 11.3%. High genetic diversity between the Chiang Mai specimens and the other South Asian matrilines suggest it could be a new species, which we describe below. 

**Table 2:** Matrix of uncorrected K2P distances among partial 16S rRNA gene sequences of members of *Fejervarya* (GenBank accession numbers follow species names)

Species	##	1	2	3	4	5	6	7	8	9	10	11	12	13
*F. syhadrensis* (AB488892)	1	-												
*F. granosa* (AB488895)	2	0. 6	-											
*F. pierrei* (AB488888)	3	0. 6	0. 4	-										
*F. chiangmaiensis* **sp. nov**. (KIZ024057)	4	6.7	6.4	6.0	-									
*F. chiangmaiensis* **sp. nov**. (KIZ024126)	5	6.7	6.4	6.0	0.0	-								
*F. kudremukhensis* (AB488898)	6	9.2	9.4	9.0	9.0	9.0	-							
*F. nilagirica* (AB167949)	7	9.2	9.4	9.0	9.0	9.0	0.0	-						
*F.* cf. *syhadrensis* (AB488893)	8	8.8	9.0	8.6	8.8	8.8	8.5	8.5	-					
*F. sahyadris* (AB530605)	9	9.0	9.3	8.8	9.0	9.0	8.1	8.1	1.2	-				
*F. caperata* (AB488894)	10	9.2	9.4	9.5	9.2	9.2	8.3	8.3	6.2	6.5	-			
*F. greenii* (AB488891)	11	10.0	10.3	10.3	9.7	9.7	9.7	9.7	8.3	8.6	7.6	-		
*F. kirtisinghei* (AY014380)	12	9.7	10.0	10.0	9.8	9.8	9.7	9.7	8.1	8.6	7.6	4.0	-	
*F. rufescens* (AB167945)	13	12.6	13.3	12.8	11.3	11.3	11.4	11.4	11.6	11.1	10.6	9.9	11.1	-
*F. gomantaki* (KR78086)	14	10.3	10.1	9.5	9.3	9.3	10.9	10.9	4.6	4.6	8.5	11.1	11.1	14.8

**Figure 4:**
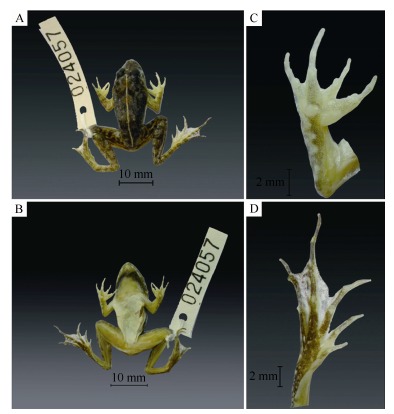
Dorsal (A) and ventral views (B) (scale bar, 10 mm); Ventral views of the right hand (C) and foot (D) (scale bar, 2 mm) of the male holotype of *Fejervarya chiangmaiensis* **sp. nov**. (KIZ024057) after preservation

**Figure 5:**
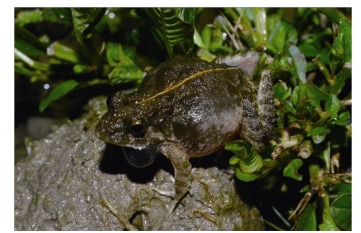
Life photo of *Fejervarya chiangmaiensis* **sp. nov**. *in situ*; calling adult male paratype KIZ024056 from type locality

***Fejervarya chiangmaiensis*** **sp. nov**. (Figure 4, 5)

**Holotype.** Adult male (KIZ024057), from Ban Monjong, Omkoi District, Chiang Mai Province, Thailand (N17°28'16.93", E98°27'28.26", 460 m a.s.l.), collected by Chatmongkon Suwannapoom on 30 June, 2013.

**Paratypes.** Eleven males KIZ024053-56, KIZ024058, KIZ024126, KIZ024096-100; collected by Chatmongkon Suwannapoom, Zhiyong Yuan, and Fang Yan; other data same as the holotype. 

** Diagnosis. **The new species assigns to *Fejervarya* on the basis of its position in the matrilineal phylogeny (Figure 3) and the following morphological characteristics: (1) slightly pointed snout; (2) comparatively poorly developed foot webbing; (3) lateral line system in adult absent; (4) characteristic "*Fejervarya*"-lines present; (5) femoral glands absent; (6) tympanum comparatively small; and (7) tibia length slightly more than a half of SVL. The new species is characterized by a combination of the following morphological characteristics: (1) small-size (males mean SVL 26.3-29.1 mm; *n*=12) (Table 3); (2) head length greater than head width; (3) tympanum small, discernible but unclear; (4) slightly elongated cylindrical internal metatarsal tubercle; (5) relative finger length (from longest to shortest) when addressed: Ⅱ<Ⅳ<Ⅰ<Ⅲ; (6) webbing formula on foot=Ⅰ 1-2Ⅱ 1-2½ Ⅲ 2-3 Ⅳ 3-1 Ⅴ; (7) cream vertebral line usually present medially lasting from between the eyes to the vent; males with paired dark vocal sacs (Figure 4); (8) dorsal and lateral parts of head and body, including body flanks, shagreened; posterior part of dorsum with distinct, elongate, glandular warts, continuing on dorsal surface of legs and arms; (9) dorsal skin showing rare, small, longitudinal folds arranged in series; and (10) advertisement call consisting of a long series of partially pulsed notes, each of which lasts 3.0±0.4 s, with 9-12 pulses/call (average 11.2±1.8), note interval of 1.8±0.6 s, and dominant frequency of 2.0 kHz.

**Table 3:** Measurements of type specimens (mm) (*n*=12 in males) of *Fejervarya chiangmaiensis* **sp. nov**.

Voucher number	**KIZ (Holotype) ** **024057**	KIZ 024054	KIZ 024097	KIZ 024098	KIZ 024126	KIZ 024100
Sex	male	male	male	male	male	male
SVL	26.3	29.1	28.8	26.9	27.9	29.1
HL	11.3	11.9	12.1	10.8	12.0	11.9
S-NL	2.2	2.5	2.6	2.3	2.5	2.6
N-EL	2.5	2.9	2.6	2.6	2.7	2.7
SL	4.8	4.7	5.0	4.7	5.2	5.3
EL	3.8	3.8	3.8	3.5	4.3	3.6
T-ED	2.0	2.3	2.8	2.0	2.6	2.6
HW	10.1	11.2	11.0	10.0	10.2	10.6
IND	2.5	2.7	2.9	2.5	2.8	2.7
IOD	1.9	2.1	1.5	1.9	1.7	1.7
UEW	3.4	3.4	4.0	3.5	3.6	3.2
FLL	18.0	19.8	19.1	16.6	18.5	18.0
LAL	11.6	12.1	11.0	10.6	12.1	11.6
FFL	5.0	5.2	6.0	4.9	4.9	5.3
HLL	47.7	51.2	50.0	47.2	48.8	49.9
TL	14.7	14.9	15.0	14.3	14.9	15.7
FL	14.4	15.7	15.8	14.3	15.7	15.7
IMTL	1.5	1.6	1.6	1.4	1.5	1.5
Ⅰ-Ⅳ FL	Ⅱ–Ⅳ–Ⅰ–Ⅲ	Ⅱ–Ⅳ–Ⅰ–Ⅲ	Ⅱ–Ⅳ–Ⅰ–Ⅲ	Ⅱ–Ⅳ–Ⅰ–Ⅲ	Ⅱ–Ⅳ–Ⅰ–Ⅲ	Ⅱ–Ⅳ–Ⅰ–Ⅲ
Ⅰ-Ⅴ TOEL	Ⅰ–Ⅱ–Ⅴ–Ⅲ–Ⅳ	Ⅰ–Ⅱ–Ⅴ–Ⅲ–Ⅳ	Ⅰ–Ⅱ–Ⅴ–Ⅲ–Ⅳ	Ⅰ–Ⅱ–Ⅴ–Ⅲ–Ⅳ	Ⅰ–Ⅱ–Ⅴ–Ⅲ–Ⅳ	Ⅰ–Ⅱ–Ⅴ–Ⅲ–Ⅳ
Voucher number	KIZ 024055	KIZ 024058	KIZ 024099	KIZ 024096	KIZ 024053	KIZ 024056
Sex	male	male	male	male	male	male
SVL	28.2	26.9	28.2	29.1	29.1	27.6
HL	11.0	10.3	11.6	11.1	11.9	11.9
S-NL	2.2	2.3	2.4	2.5	2.7	2.5
N-EL	2.6	2.7	2.6	3.0	3.0	2.9
SL	4.8	5.0	5.0	5.4	5.7	5.4
EL	3.4	3.4	3.6	4.1	4.1	3.8
T-ED	2.0	2.7	2.6	2.3	2.7	2.2
HW	9.9	9.3	9.9	10.4	10.9	10.4
IND	2.2	2.2	2.5	2.8	2.9	2.6
IOD	1.8	1.9	1.7	2.1	2.4	2.1
UEW	3.2	3.5	3.6	3.7	3.9	3.3
FLL	17.3	17.2	16.9	18.6	20.6	17.2
LAL	11.8	11.5	12.0	12.4	12.6	11.3
FFL	5.1	4.7	5.3	6.0	5.6	4.6
HLL	47.2	46.8	45.8	51.5	50.9	47.6
TL	14.2	14.8	12.4	16.0	15.4	14.7
FL	15.6	14.4	15.5	15.8	16.2	14.1
IMTL	1.5	1.4	1.5	1.7	1.7	1.4
Ⅰ-Ⅳ FL	Ⅱ–Ⅳ–Ⅰ–Ⅲ	Ⅱ–Ⅳ–Ⅰ–Ⅲ	Ⅱ–Ⅳ–Ⅰ–Ⅲ	Ⅱ–Ⅳ–Ⅰ–Ⅲ	Ⅱ–Ⅳ–Ⅰ–Ⅲ	Ⅱ–Ⅳ–Ⅰ–Ⅲ
Ⅰ-Ⅴ TOEL	Ⅰ–Ⅱ–Ⅴ–Ⅲ–Ⅳ	Ⅰ–Ⅱ–Ⅴ–Ⅲ–Ⅳ	Ⅰ–Ⅱ–Ⅴ–Ⅲ–Ⅳ	Ⅰ–Ⅱ–Ⅴ–Ⅲ–Ⅳ	Ⅰ–Ⅱ–Ⅴ–Ⅲ–Ⅳ	Ⅰ–Ⅱ–Ⅴ–Ⅲ–Ⅳ

Abbreviations are listed in Material and Methods. KIZ=Kunming Institute of Zoology.

**Description of holotype.** (all measurements in mm; see Table 3): Adult male (KIZ024057; Figure 4). Small-sized frog specimen with SVL=26.3 mm, body habitus moderately stout (Figure 4A). 

**Head. **Head of moderate size, head longer (HL 11.3 mm) than wide (HW 10.1 mm; HW/HL ratio 0.9), convex (Figure 4A). Snout more or less pointed from above; length (SL 4.8 mm) longer than horizontal diameter of eye (EL 3.8 mm) and interorbital distance (IOD 1.9 mm). Snout relatively distinct, protuberant; loreal region concave, angle to upper surface of snout rather vertical; canthus rostralis not sharp, rounded. Interorbital space slightly convex, much narrower (IOD 1.9 mm) than upper eyelid (UEW 3.4 mm) and narrower than internarial distance (IND 2.5 mm). Nostrils rounded, with a distinct flap of skin laterally, nostril slightly closer to snout than to eye (S-NL: 2.2 mm; N-EL: 2.5). Eyes comparatively small, protuberant; EL 33.5% of HL; upper eyelid with minute granules, pupil horizontal. Tympanum (TYD 1.4 mm) visible, but poorly distinct, rounded; slightly more than the half of eye diameter (TD 53.0% of ED), tympanum-eye distance (TYE 2.0 mm) half its diameter. Pineal ocellus indistinct. Choanae triangular, rounded; vomerine ridge absent, vomerine teeth in two oblique lines between choanae, beginning at anterior border of choanae, slightly extending beyond its posterior border. Tongue rather large, cordate, notably forked with two projections at tip; median lingual process absent; tooth like projections on maxilla absent.

**Forelimbs. **Lower arm short, rather strong (LAL 11.6 mm), 64.3% of forelimb length (FLL 18 mm). Fingers short, thin, without dermal fringe; webbing absent, finger tips bluntly rounded and not enlarged to disks (Figure 4C). Relative finger lengths from shortest to longest: Ⅱ<Ⅳ<Ⅰ<Ⅲ (Ⅰ: 5.5 mm; Ⅱ: 5.1 mm; Ⅲ: 6.6 mm; Ⅳ: 5.5 mm). Subarticular tubercles prominent, rounded, single, all present; a single palmar (thenal) tubercle large, well-developed, rounded. Prepollex oval, distinct; supernumerary tubercles absent. 

**Hindlimbs.** Hindlimbs comparatively long, HLL (47.7 mm), about 1.8 times that of SVL (26.3 mm). Tibia (TL 14.7 mm) slightly longer than femur and subequal to foot length (FL 14.4 mm). Toes long, thin, toe tips blunt, slightly rounded, not enlarged to disks (Figure 4D). Relative toe lengths from shortest to longest: Ⅰ<Ⅱ<Ⅴ<Ⅲ<Ⅳ (Ⅰ: 5.90 mm; Ⅱ: 7.46 mm; Ⅲ: 10.71 mm; Ⅳ: 13.5 mm; Ⅴ: 9.5 mm). Subarticular tubercles prominent, elongated oval-shaped, protuberant, simple, all present. Inner metatarsal tubercle prominent, long and slightly compressed laterally (IMT: 1.5), 4 times the length of toe Ⅰ. Foot webbing small, webbing formula Ⅰ 1-2Ⅱ 1-2½Ⅲ 2-3 Ⅳ 3-1 Ⅴ (Figure 4C, D). Dermal fringe along toe Ⅴ absent. Inner tarsal ridge present, flat. Outer metatarsal tubercle present, prominent, elongated; supernumerary tubercles absent; tarsal tubercle absent.

**Skin and skin glands. **Snout smooth with rare indistinct dermal granules, nares with low dermal flaps, small tubercles on upper eyelid. Dorsal and lateral surfaces of head and body, including body flanks, shagreened, posterior part of dorsum with distinct, round glandular warts, continuing on dorsal surfaces of legs and arms; Dorsal skin showing rare, small, longitudinal dermal ridges arranged in series. Anterodorsal part of thigh, anal region, dorsal surface of tibia, and tarsus with small granules; lateral sides of body, ventral surfaces of body and limbs smooth. Lateral-dorsal folds absent; lateral line system absent; "*Fejervarya*"-line present; supra-tympanic fold distinct, running from the posterior corner of eye backwards and downwards towards the incursion of the forelimb. Discernable macroglands, in particular rictal gland, absent.

**Male secondary sexual characters.** White nuptial pas present. Vocal sacs present, unique subgular pouch appear subdivided when inflated (Figure 5); pair of rounded openings in posterior part of mouth floor. No other discernable male secondary characters.

**Coloration of adult in life. **Dorsal ground color varies from dark green to dark brown; lateral sides greyish, ventral surfaces of body, head, and limbs whitish-cream. Marginal sides of gular area dark-greyish to blackish forming an M-shaped band (when inflated, vocal sac looks blackish; Figure 5). Transverse blackish-green to dark brown bands present on the dorsal surfaces of the thigh, tibia, and tarsus (Figure 5). Dorsal surfaces of fingers and toes greyish with indistinct transverse bands. Three to four dark brownish irregular blotches on each side of the upper jaw. Lateral sides of body with irregular grey-greenish and whitish blotches; dark greenish blotches get bigger towards the axilla and groin. Thin orange-yellowish mid-dorsal vertebral stripe runs from the anterior part of the interorbital space to the vent. Numerous fused irregular spots on the posterior surface of thigh dark green with thin dark brown reticulations in-between. Tympanum unclear dark green with darker circle in center, lower part of tympanum greyish. Iris greenish-bronze with dark reticulation; pupil horizontal. 

**Coloration in preservative.** In alcohol the pattern described above was not obviously changed, although was slightly faded (Figure 4A). The yellowish tint faded the most, with greenish colors on the dorsal surfaces appearing brownish or gray-brownish (Figure 4A); the ventral sides look much lighter than in life (Figure 4B); the belly appeared whitish. Dorsum turned greyish brown with many large dark-gray to black spots, thin mid-dorsal stripe turned whitish, running from the interorbital space to the vent. Lateral with many small blackish dots, ventral immaculate except for a dark-grey M-shaped band across the gular area (Figure 4B). Transverse black bands on the dorsal surfaces of thigh, tibia, and tarsus were easily discernable. 

**Variation.** Variation in meristic and morphometric characters among the type series are shown in Table 3. Individuals of the type series are generally similar in appearance, but show certain variation in coloration and dorsal pattern. Among the studied types, we found variation in the degree of development of mid-dorsal vertebral light line. Paratypes resemble the holotype in all aspects of morphology except for KIZ024053, KIZ024054 and KIZ024100, which do not have the thin whitish mid-dorsal stripe from tip of between eyes to vent (27%). In KIZ024098, the mid-dorsal line is broken in the scapular area. Variation in dorsal coloration is also observed: the head might be light dark green to dark brown and the dorsum is greyish brown with many large black spots. 

**Advertisement call.** Calls were recorded at an air temperature of 26.0℃. This species had both short and long calls, though the latter were not always emitted (Figure 2). The shorter call consisted of a series of pulsed notes. Each of these notes lasted 3.0±0.4 s and was composed of 9-12 pulses/call (average 11.2±1.8). The note interval was 1.81±0.598 s, the dominant frequency was 2.0±0.03 kHz, and the second harmonic was about 3 857±0.036 kHz. The call had a slight frequency modulation.

**Etymology.** The specific epithet *chiangmaiensis* is a Latinized adjective derived from the name of Chiang Mai Province, Thailand. We suggest the common English name "*Chiang Mai Rain-Pool Frog*" and vernacular name in Thai "*Kob-Nonglek Chiang Mai*", taken from "*Kob*" for frog, "*Nonglek*" for small swamp, "*Chiang Mai*" for Chiang Mai Province, Thailand.

**Distribution and habitat.** *Fejervarya chiangmaiensis* **sp. nov**. is, to date, known only from a single locality, encompassing a lowland farm in Ban Monjong, Omkoi District, Chiang Mai, northern Thailand (N17°28'16.93", E98°27'28.26"; 460 m a.s.l.). The frogs were found calling on clods of dirt in rice fields at night during rainfall; it appears that the species inhabits disturbed habitats, including agricultural areas. Females of the new species remain unknown. The new species was found in sympatry with *F*. *limnocharis*, *Occidozyga lima*, and *Hoplobatrachus rugulosus*.

### Comparisons with other congeners

*Fejervarya chiangmaiensis* **sp. nov**. can be distinguished from large- and medium-sized members of *Fejervarya* in external morphology, coloration, and acoustics. *Fejervarya chiangmaiensis* **sp. nov**. can be distinguished from members of the sister matriline, comprised of *F*. *granosa* (central Western Ghats, India), *F*. *pierrei* (Nepal, Bangladesh and E India), and *F*. *syhadrensis* (India, Pakistan, Bangladesh, Sri Lanka, and Nepal). The SVL of male *F*. *chiangmaiensis* **sp. nov**. (26.3-29.1 mm) overlaps with male *F*. *granosa* (29.1 mm; Kuramoto et al., 2007), *F*. *pierrei* (24.7-41.2 mm; Dubois, 1975), and *F*. *syhadrensis* (22-36 mm; Howlader, 2011b). However, the new species clearly differs in the dominant frequency of its advertisement call, which is higher (2.0 kHz) than that in *F*. *granosa* (1.7 kHz), but much lower than that in *F*. *pierrei* and *F*. *syhadrensis* (4.2 kHz and 2.7-4.1 kHz) (Kuramoto et al., 2007; Purkayastha & Matsui, 2012). Moreover, the new species can be further distinguished from *F*. *granosa* and *F*. *pierrei* by dorsum shagreened with rare low dorsal ridges and distinct glandular warts in the posterior part of the dorsum, and by moderately stout body habitus (vs. dermal ridges on the back well-pronounced, generally short or rounded and body shape relatively thick in *F*. *granosa*, Kuramoto et al., 2007; and vs. dorsum highly tuberculated, habitus stocky in *F*. *pierrei*, Dubois, 1975). The new species can be further distinguished from *F*. *pierrei* by relative finger lengths, with the second finger being shorter than the fourth finger (Ⅱ<Ⅳ<Ⅰ<Ⅲ in *F*. *chiangmaiensis* **sp. nov**. vs. Ⅱ=Ⅳ<Ⅰ<Ⅲ in *F*. *pierrei*; Howlader, 2011b). *Fejervarya chiangmaiensis* **sp. nov**. can be further differentiated from *F*. *syhadrensis* by head width less than head length, HW/HL rate 0.9 (vs. head broader than long, HW/HL rate 1.0 in *F*. *syhadrensis*, see Kuramoto et al., 2007) and by relative finger lengths (Ⅱ<Ⅳ<Ⅰ<Ⅲ in *F*. *chiangmaiensis* **sp. nov**. vs. Ⅰ=Ⅱ<Ⅳ<Ⅲ in *F*. *syhadrensis*; Howlader, 2011b). 

The body size of male *F*. *chiangmaiensis* **sp. nov**. differs from that of other species of *Fejervarya*. The new species (males 26.3-29.1 mm; Thailand) is smaller than *F*. *sengupti* (males 33.4-35.7 mm; Maghalaya, India; Purkayastha & Matsui, 2012) and much smaller than *F*. *kudremukhensis* (males 40.8-43.3 mm; Western Ghats, India; Kuramoto et al., 2007). The new species also clearly differs by the dominant frequency of the advertisement call, which is lower than that in both *F*. *sengupti* and *F*. *kudremukhensis* (2.0 kHz vs. 3.3 kHz and 3.6 kHz, respectively; as reported by Kuramoto et al., 2007; Purkyastha & Matsui, 2012). *Fejervarya chiangmaiensis* **sp. nov**. can be further diagnosed from *F*. *sengupti* and *F*. *kudremukhensis* by dorsum shagreened with granules in the posterior part, and by head width less than head length, HW/HL rate 0.89 (vs. dorsum densely granulated with dermal transversal folds, and head notably wider than long, HW/HL rate 1.2 in *F*. *sengupti*, see Purkyastha & Matsui 2012; and vs. dorsum with a few short dermal ridges and interrupted reversed V-shaped ridge in the scapular area, and head wider than long, HW/HL rate 1.1 in *F*. *kudremukhensis*, data for males, see Kuramoto et al., 2007). 

Although comparative data are limited, the SVL of *F*. *chiangmaiensis* **sp. nov**. overlaps with the SVL of male *F*.*nepalensis* (23.0-37.8 mm, Nepal, NE India, Bhutan, Bangladesh; see Dubois, 1975). However, the new species clearly differs from the latter by relative finger length: Ⅱ<Ⅳ<Ⅰ<Ⅲ and shagreened dorsum vs. Ⅱ<Ⅰ<Ⅳ<Ⅲ and warted dorsum in *F*. *nepalensis*. 

The following species of *Fejervarya* have greater male SVL values than that of *F*. *chiangmaiensis* **sp. nov**: *F*. *mysorensis* from India (37.0 mm: Dutta, 1997 as *Limnonectes*), *F*. *teraiensis* from Nepal (40.1-50.5 mm; Matsui et al., 2007), and *F*. *murthii* (35.0 mm: Dutta, 1997 as *Limnonectes*) and *F*. *nilagirica* (34.7-42.2 mm), two endemic species from India. 

Although *F*. *asmati* from Bangladesh, *F*. *keralensis* from India, and *F*. *brevipalmata* from India overlap with the new species in body size (SVL 29.1-33.4 mm: Howlader, 2011a; 21.2-47.0 mm: Dutta, 1997, and 28.3-59.8 mm: Dutta, 1997 as *Limnonectes*, respectively), the presence of these species in northern Thailand appears to be highly improbable (see Bauer et al., 1995; Boulenger, 1905; Choudhury et al., 2001; Howlader, 2011b; Peters, 1871; Purkayastha & Matsui, 2012). The new species can be further differentiated from *F*. *asmati* by snout pointed in lateral view (vs. snout almost rounded in lateral view), by skin on dorsum shagreened without dermal folds in shape of inversed V (vs. skin on dorsum with transverse elongated ridges forming inversed V-pattern), by coloration of vocal sacs in breeding males forming a dark M-shaped pattern on the gular area (vs. characteristic butterfly-shaped spot on throat in males), and by the dominant frequency of the advertisement call, which is lower than that in *F*. *asmati* (2.0 kHz vs. 4.1-5.1 kHz) (Howlader, 2011b). The new species can be further distinguished from *F*. *keralensis* by having a comparatively larger head (HL/SVL 0.4 in *F*. *chiangmaiensis* ****sp. nov**** vs. 0.4 in *F*. *keralensis* males) and larger eyes (EL/SVL 0.1 in *F*. *chiangmaiensis* **sp. nov** vs. 0.1 in *F*. *keralensis*, data for males, see Kuramoto et al., 2007) and less developed small webbing; web formula: Ⅰ 1-2Ⅱ 1-2-Ⅲ 2-3 Ⅳ 3-1 Ⅴ (vs. wide almost complete webbing in *F*. *keralensis*; web formula: Ⅰ 1-2 ⅡII 2-1 Ⅲ 1-1 Ⅳ 1-1 Ⅴ, Kuramoto et al., 2007).

The three other species of *Fejervarya* from the Western Ghats in southern India, *F*. *nilagirica*, *F*. *caperata*, and *F*. *mudduraja*, can be also differentiated from the new species on the basis of body size and proportions and by presence of warts and dermal ridges on the dorsum (Kuramoto et al., 2007). *Fejervarya nilagirica* is a large-bodied species and can be easily distinguished from the small-bodied *F*. *chiangmaiensis* **sp. nov**. (male SVL 26.3-29.1); and further distinguished by the numerous warts and dermal ridges on the dorsum (vs. smooth to shagreened dorsum with glandular warts in posterior part in *F*. *chiangmaiensis* **sp. nov**.) and by relatively smaller eyes, EL/SVL min 0.1-0.2 in the new species. Although *F*. *caperata* overlaps with *F*. *chiangmaiensis* **sp. nov**. in SVL (mean SVL being 33 mm in females and 29 mm in males), it can be distinguished by its relatively slender body habitus (vs. moderately stout body habitus in the new species), by long dermal ridges on the dorsum forming four longitudinal lines (vs. smooth to shagreened dorsum with glandular warts in posterior part in *F*. *chiangmaiensis* **sp. nov**.), and by relative finger lengths Ⅳ<Ⅱ<Ⅰ<Ⅲ (vs. relative finger lengths Ⅱ<Ⅳ<Ⅰ<Ⅲ in the new species). *Fejervarya mudduraja* can be distinguished by its large body size, with mean SVL of females being 45 mm (no information on male SVL available) (vs. small body size in *F*. *chiangmaiensis* **sp. nov**., SVL 26.3-29.1), presence of long dermal ridges on the back arranged into four longitudinal lines (vs. smooth to shagreened dorsum with glandular warts in posterior part in *M*. *chiangmaiensis* **sp. nov**.), and by head wider than long, HW/HL=1.1 (vs. head width less than head length, HW/HL=0.9 in *F*. *chiangmaiensis* **sp. nov**.).

*Fejervarya murthii* (Tamil Nadu, Karnataka, south India) can be distinguished by the presence of two triangular patches bearing pearl-like papillae on the breast in males, and presence of the papillae in the anterior part of the lower jaw (Pillai, 1979) (vs. no such discernible papillae in *F*. *chiangmaiensis* **sp. nov**.).

The new species can be easily diagnosed from *F*. *teraiensis* inhabiting Nepal and NE India by its smaller body size (SVL 26.3-29.1 vs. SVL 37.8-44.1 in males of *F*. *teraiensis*; Howlader, 2011b), relative finger lengths (Ⅱ<Ⅳ<Ⅰ<Ⅲ in *F*. *chiangmaiensis* **sp. nov**. vs. Ⅱ<Ⅳ<Ⅰ<Ⅲ in *F*. *teraiensis*; Howlader, 2011b) and head width less than head length, HW/HL rate 0.9 (vs. head width almost equal to head length, HW/HL rate 1.0 in *F*. *teraiensis*).

*Fejervarya rufescens* from southern India can be distinguished by having stocky body habitus (vs. moderately stout habitus in *F*. *chiangmaiensis* **sp. nov**.), highly tuberculated skin on the dorsum with pronounced transverse dermal ridges forming an inverse V-pattern (vs. shagreened dorsum with rare low dorsal ridges in in *F*. *chiangmaiensis* **sp. nov**.), and reddish coloration on the dorsum in breeding males (vs. grayish or greenish dorsal coloration in breeding males of *F*. *chiangmaiensis* **sp. nov**.). Sri Lankan *F*. *kirtisinghei* and *F*. *greenii*, can be easily differentiated by the dorsum covered with well-developed long continuous dermal ridges (vs. shagreened dorsum with rare low dorsal ridges, never forming continuous rows in *F*. *chiangmaiensis* **sp. nov**.).

Comparison of *F*. *chiangmaiensis* **sp. nov**. with certain dicroglossid species reported for the region is complicated due to their unclear taxonomic status. Locality of *F*. *brevipalmata*, originally designated as "Pegu, Myanmar", appears to be uncertain. This species likely occurs in the Western Ghats of southern India. Its taxonomic status is unclear (AmphibiaWeb, 2016; Boulenger, 1920). Furthermore, *F*. *sauriceps* and *F*. *parambikulamana*, two endemic species from Kerala and Karnataka of southern India, are known only from holotypes that appear to be lost. *Fejervarya sauriceps* is supposed to differ from other known *Fejervarya* by a very small tongue, unique triangular pit on the snout, a brown venter, and wide interorbital width (more than twice the upper eyelid width), whereas *F*. *parambikulamana* is diagnosed by smooth dorsum and comparatively longer legs (Kuramoto et al., 2007); both differ from *F*. *chiangmaiensis* **sp. nov**. Taxonomic validity and systematic status of these species requires further investigation (AmphibiaWeb, 2016; Frost, 2016) and some researchers doubt their validity (Matsui et al., 2007; Purkayastha & Matsui, 2012). Similar concerns have been raised on some other dicroglossid frog species known in regions nearby, in particular *F*. *altilabris* (Blyth) from Myanmar, *F*. *frithii* (Theobald) from Bangladesh, and *F*. *assimilis* (Blyth) and *F*. *brama* (Lesson) from India (Matsui et al., 2007). *Fejervarya chiangmaiensis* **sp. nov**. can be further differentiated by its smaller body size from *F*. *orissaensis* (Orissa, India; Dutta, 1997) (males 26.3-29.1 mm vs. 36.2-47.2 mm in *F*. *orissaensis* Dutta (1997) as *Limnonectes*). Furthermore, *F*. *chiangmaiensis* **sp. nov**. can be distinguished from the two other miniaturized species of *Fejervarya* (*F*. *sahyadris and F*. *chilapata*) by a larger SVL in males (male of *F*. *chiangmaiensis* **sp. nov**., mean SVL=28.1±1.0; *n*=12 vs. males of *F*. *sahyadris and F*. *chilapata*, mean SVL=18.4±6.0 mm; *n*=10, SVL=20.0±7.0 mm; *n*=8, respectively), by lacking a white stripe on the upper lip (vs. white stripe present in *F*. *sahyadris and F*. *chilapata*), by lacking light dorsolateral lines (vs. light dorsolateral lines present in *F*. *sahyadris* and *F*. *chilapata*), by having shagreened dorsal and lateral surfaces of head and body including body flanks, posterior part of dorsum with distinct, round glandular warts, continuing on dorsal surfaces of legs and arms (vs. dorsal and lateral parts of head and body smooth; posterior part of back with indistinct, glandular warts in *F*. *sahyadris* and *F*. *chilapata*), and by different advertisement call (*F*. *sahyadris *(3.6-4.4 kHz) and *F*. *chilapata* (2.0 kHz) have a higher dominant frequencies than that of *F*. *chiangmaiensis* **sp. nov**. (2.0 kHz)). 

Numerous differences in morphology, coloration, acoustics, and mtDNA gene sequences give support to recognizing the specimens collected in Chiang Mai Province of Thailand as a new species. Accordingly, description is necessary to accurately document the anuran biodiversity of Thailand.
